# Pendant Modification of Poly(methyl methacrylate) to Enhance Its Stability against Photoirradiation

**DOI:** 10.3390/polym15142989

**Published:** 2023-07-09

**Authors:** Shaymaa Sansul, Emad Yousif, Dina S. Ahmed, Gamal A. El-Hiti, Benson M. Kariuki, Hassan Hashim, Ahmed Ahmed

**Affiliations:** 1 Department of Chemistry, College of Science, Al-Nahrain University, Baghdad 64021, Iraq; st.shaimaa.hassan.sansol@ced.nahrainuniv.edu.iq (S.S.); emad.yousif@nahrainuniv.edu.iq (E.Y.); 2 Department of Chemical Industries, Institute of Technology-Baghdad, Middle Technical University, Baghdad 10074, Iraq; dina_saadi@mtu.edu.iq; 3 Department of Optometry, College of Applied Medical Sciences, King Saud University, Riyadh 11433, Saudi Arabia; 4 School of Chemistry, Cardiff University, Main Building, Park Place, Cardiff CF10 3AT, UK; kariukib@cardiff.ac.uk; 5 Department of Physics, College of Science, Al-Nahrain University, Baghdad 64021, Iraq; hassan.hashim@nahrainuniv.edu.iq; 6 Polymer Research Unit, College of Science, Al-Mustansiriyah University, Baghdad 10052, Iraq; dr_ahmedabd@uomustansiriyah.edu.iq

**Keywords:** polymeric material pendant modification, radicals scavenger, poly(methyl methacrylate), ultraviolet irradiation, surface morphology, weight loss, decomposition rate constant

## Abstract

Photostabilization of functional polymeric materials is important for protection against aging and ultraviolet (UV) irradiation. There is, therefore, the impetus to modify polymers to increase their resistance to photodegradation and photooxidation on extended exposure to UV light in harsh conditions. Various polymeric additives have been designed and synthesized in recent years, and their potential as photostabilizers has been explored. Reported here is the effect of pendant functionalization of poly(methyl methacrylate) (PMMA) through organometallic moiety incorporation into the polymer’s backbone. The reaction of PMMA with ethylenediamine leads to the formation of an amino residue that can react with salicylaldehyde to produce the corresponding Schiff base. Adding metal chlorides (zinc, copper, nickel, and cobalt) led to the formation of organometallic residues on the polymeric chains. Thin films of modified and unmodified PMMA were produced and irradiated with UV light to determine the effect of pendant modification on photostability. The photostabilization of PMMA was assessed using a range of methods, including infrared spectroscopy, weight loss, decomposition rate constant, and surface morphology. The modified PMMA incorporating organic Schiff base metal complexes showed less photodecomposition than the unmodified polymer or one containing the Schiff base only. Thus, the metals significantly reduced the photodegradation of polymeric materials. The polymer containing the Schiff base-cobalt unit showed the least damage in the PMMA surface due to photoirradiation, followed by those containing nickel, zinc, and copper, in that order.

## 1. Introduction

Polymers have wide-ranging applications due to their variety of properties, low manufacturing cost, and ability to be molded into various shapes [1]. To enhance the properties of polymers, functionalization techniques are utilized through physical, chemical, and mechanical processes [2]. Physical processes entail surface modification or coating deposition, which are simple. Chemical processes, on the other hand, modify polymeric chains by attaching molecules with specific functional groups through covalent bonds. Meanwhile, mechanical methods create rough polymer microstructures on surfaces [2]. The properties of polymeric materials are commonly improved through judicious blending and doping [3]. These processes can lead to key alterations in the structure, surface morphology, physical properties, and performance of polymeric materials [4]. The successful doping of polymeric materials with organic motifs containing metals can improve corrosion resistance, durability, and stability with aging [5]. Progressive improvements have been made, but continual research is still necessary to produce more efficient polymeric composites suitable for demanding applications [6,7]. Factors that affect the doping process include miscibility, dispersion, phase segregation, particle size, and the crystalline nature of the additives used [8].

Poly(methyl methacrylate) (PMMA) has many applications, either in pure form or as bends or composites [9,10]. PMMA is used, for example, in optical devices (e.g., lenses), medical equipment and devices (e.g., dental equipment, surgical instruments, and prosthetic implants), drug delivery, and photoresists [11,12,13]. PMMA has a unique combination of characteristics, such as high transparency, low density, good mechanical and physical properties, cost-effectiveness, durability, and ease of manipulation. However, PMMA also has disadvantages, including suffering from photodegradation and photodecomposition in harsh conditions (e.g., high levels of temperature and humidity in the presence of oxygen) [14]. Irradiation of PMMA with UV leads to destructive effects such as discoloration, reduction of mechanical strength, and formation of holes and cracks [15]. The photodegradation of PMMA occurs through bond cleavage and the formation of shorter polymeric chains. The mechanism of photodegradation involves chain scission and the production of free radicals. A direct relationship has been established between the level of damage in polymeric materials and the length of the irradiation period [16,17,18]. Thus, there is a motivation for mixing polymeric materials with UV inhibitors (e.g., metallic complexes, organics, fibers, and pigments) or structural modification to reduce the damage due to photodegradation [19]. The photostabilization of PMMA depends on various factors, such as particle size and the nature and properties of the additives [20,21,22,23,24,25,26,27,28,29].

Adding UV photostabilizers to polymers inhibits cross-linking, branching, and bond cleavage when exposed to light [30,31,32]. Inorganic additives (e.g., metal oxides) tend to be unevenly distributed within the polymeric matrix, and their efficiency mainly depends on their particle size and concentration [33,34,35]. Applying organic additives (e.g., benzotriazoles, phenyl benzoate, or hydroxybenzophenone) can also be limited due to their volatility, incompatibility, toxicity, and leakage [36,37,38]. Thus, research into ways to overcome the various limitations associated with UV additives continues. The design and use of new additives to stabilize polymers have been active in recent years [39,40,41]. As part of our interest in using additives in stabilizing polymeric materials [42,43,44,45,46], we have shown that PMMA can be stabilized by adding metal complexes containing naphthalene [47]. Reported here is current research showing successful pendant functionalization of PMMA through the incorporation of Schiff base metal complexes to improve photostability.

## 2. Materials and Methods

### 2.1. Materials and General

PMMA (74315 gm/mol), metal chlorides (97–98%), ethylene diamine (≥99%), salicylaldehyde (98%), and analytical grade solvents were obtained from Merck (Gillingham, UK). The ^1^H NMR spectra (400 MHz) were recorded in DMSO-d_6_ using a Bruker Avance Spectrophotometer (Tokyo, Japan).

### 2.2. Preparation of PMMA Containing Ethylene Amine

PMMA (5 g, 49 mmol) was stirred in a mixture of 2-propanol and water (1:1; 15 mL) at 25 °C for 10 min. The polymer was removed, dried, and submerged in a mixture of ethylenediamine (H_2_N(CH_2_)_2_NH_2_; 29.5 g, 490 mmol) and dimethyl sulfoxide (DMSO; 15 mL). The mixture was refluxed for 1 h with constant stirring, followed by removing the solvent. The aminated polymer was left to dry in the air for 24 h at 25 °C.

### 2.3. Preparation of PMMA Containing Schiff Base

Chloroform (CHCl_3_; 15 mL) was added to the aminated PMMA (2.5 g, 25 mmol), and the mixture was stirred for 5 min at 25 °C. Salicylaldehyde (3.7 g, 30 mmol) was then added, and the mixture was stirred at 55 °C for another 2 h. The mixture thereafter was left to dry in the air for 72 h to yield the PMMA–Schiff base blend.

### 2.4. Preparation of PMMA Containing Schiff Base and Metal

A mixture of PMMA–Schiff base (0.4 g) and appropriate metal chloride [MCl_2_, M = Zn(II), Cu(II), Ni(II), and Co; 0.1 g] in CHCl_3_ (4 mL) was sonicated for 1 h. The mixture was poured onto glass plates (thickness of approximately 40 μm) and allowed to dry (24 h at 25 °C). The films produced were left for an additional 3 h under a vacuum to remove any solvent residues.

### 2.5. Irradiation of PMMA Films

UV irradiation of PMMA films was carried out using an accelerated weather-meter QUV tester (Q-Panel Company; Homestead, FL, USA). The tester contained a stainless-steel plate with two UV fluorescent lamps (40 W; UV-B 365), one on each side. The PMMA films were irradiated at 25 °C with a UV light with an intensity of 6.2 × 10^–9^ Einstein dm^–3^ s^–1^. The films were kept at a distance of 10 cm from the tester in a vertical orientation and parallel to the fluorescent lamps. The PMMA films were rotated during the process to ensure that all sides were evenly irradiated. The films were irradiated for up to 300 h, with samples being analyzed every 50 h.

### 2.6. FTIR Spectroscopy of PMMA Films

The Fourier transform infrared (FTIR) spectra were recorded using a Shimadzu FTIR-8300 spectrophotometer (Tokyo, Japan) to monitor the increase in the intensity of the absorption band corresponding to the hydroxyl group (OH) during irradiation. The growth in the intensity of the OH absorption band (3204 cm^–1^) was compared to a reference band that irradiation has little effect on (C–H; 1443 cm^–1^ or C–C; 750 cm^–1^) [14]. The index of the OH group (I_OH_) was calculated at each irradiation time using Equation (1), based on the absorption of the OH and reference absorption bands (As and Ar, respectively) [48].

(1) Is=As/Ar

### 2.7. Weight Loss of Irradiated PMMA Films

The reduction in the weight of irradiated PMMA films was monitored to investigate the level of its photodegradation. The percentage of weight loss from PMMA films was calculated at different irradiation times using Equation (2), based on the weight of films before and after irradiation (Wo and Wt, respectively) [49].

(2) $$Weight\;loss\left(\%\right)=\frac{Wo-Wt}{Wo}\times 100$$

### 2.8. Photodecomposition Rate Constant of PMMA

The UV measurements of the PMMA film were performed using a Shimadzu spectrophotometer (Tokyo, Japan). The *k*_d_ for the irradiated PMMA films was calculated using Equation (5), which was obtained from 3 and 4 [50]. In the equations, ${x=A_{0}-A}_{t}$, *a* = the PMMA concentration pre-irradiation; *x* = the change in PMMA concentration at an irradiation time *t*; *A*_0_ = the PMMA absorption intensity at *t*_0_; *A_∞_* = the PMMA absorption intensity at *t_∞_*; and *A_t_* = the post-irradiation absorption intensity at time *t*.

(3) $$\ln\left(a-x\right)=lna-k_{d}t$$

(4) $${a-x=A_{0}-A}_{\infty }-{A_{0}+A}_{t}={A_{t}-A_{\infty }}_{}$$

(5) $${ln(A_{t}-A}_{\infty })=ln\left({A_{0}-A_{\infty }}_{}\right)-k_{d}t$$

### 2.9. Surface Morphology of Irradiated PMMA Films

The PMMA film surface damage due to irradiation was inspected using various microscopic techniques. Meiji Techno (Tokyo, Japan), SIGMA 500 VP (Carl Zeiss Microscopy; White Plains, NY, USA), and Veeco (Plainview, NY, USA) microscopes were used to record optical, scanning electron microscopy (SEM), and atomic force microscopy (AFM) images, respectively.

## 3. Results and Discussion

### 3.1. Pendant Modifications of PMMA

**Scheme 1:**
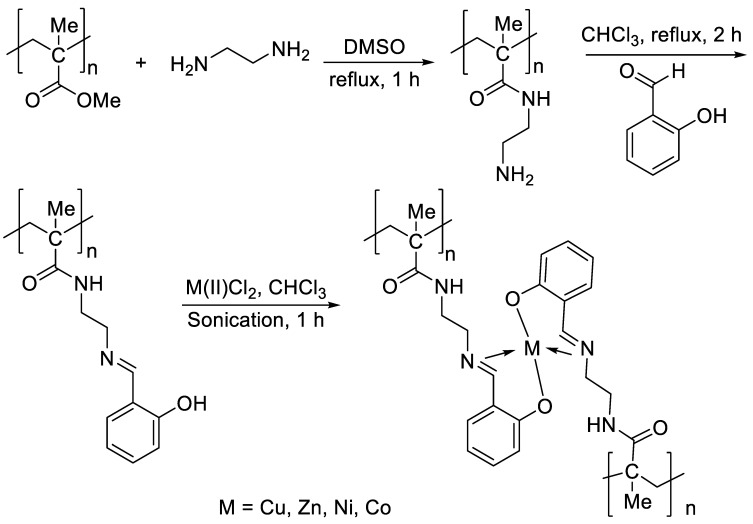
Modification of PMMA.

The route to pendant modification of PMMA is shown in Scheme 1, and is based on reported procedures [51,52,53,54]. The first stage involves the aminolysis of PMMA in boiling DMSO to enable the attachment of ethylene diamine to the polymer backbone. The mechanism for the aminolysis process involves an S_N_^2^ reaction. The second stage involves the reaction of salicylaldehyde and the amino-functionalized PMMA in boiling CHCl_3_ to form a Schiff base moiety attached to the polymer chain. Finally, the third stage involves the addition of metal chlorides under sonication to allow the formation of metal complexes of the Schiff base on the PMMA chains. The conversion to the modified PMMA was 70–77% (Table 1) based on ^1^H NMR spectroscopy.

The FTIR spectra of the modified metal complex-containing PMMA showed the presence of a sharp, intense absorption peak at 1726 cm^–1^ due to the stretching vibration of the carbonyl group (C=O) of the ester unit. The stretching vibration of the ester C–O bond appeared as a broad band in the 1141–1144 cm^–1^ region. The bending vibration of the C–H group appeared as a broad band at the 737–750 cm^–1^ region. A new absorption band appeared at the 3393 cm^–1^ region due to the NH group (ethylene diamine linkage). In addition, the formation of the Schiff base linkage was confirmed by the appearance of a strong absorption band at 1617–1631 cm^–1^ due to the CH=N bond, while the absorption band for the C–N bond appeared in the 1238–1241 cm^–1^ region. The absorption bands corresponding to the M–O bonds were in the 460–472 cm^–1^ region for the modified PMMA [55,56]. The FTIR spectra of modified and unmodified PMMA (Figures S1–S6) are shown in the Supplementary Materials. The ^1^H NMR spectra of the PMMA showed a singlet at a very low field (8.09 ppm) corresponding to the NH proton. The IR spectra showed the presence of a singlet at 8.58 ppm due to the presence of the CH=N proton. In addition, the protons corresponding to the aryl ring and methoxy group appeared at 6.86–7.73 ppm and 3.39 ppm, respectively. Clearly, the linkages containing metal complexes were attached to the PMMA chains.

### 3.2. FTIR Spectroscopy of Irradiated PMMA Films

**Figure 1:**
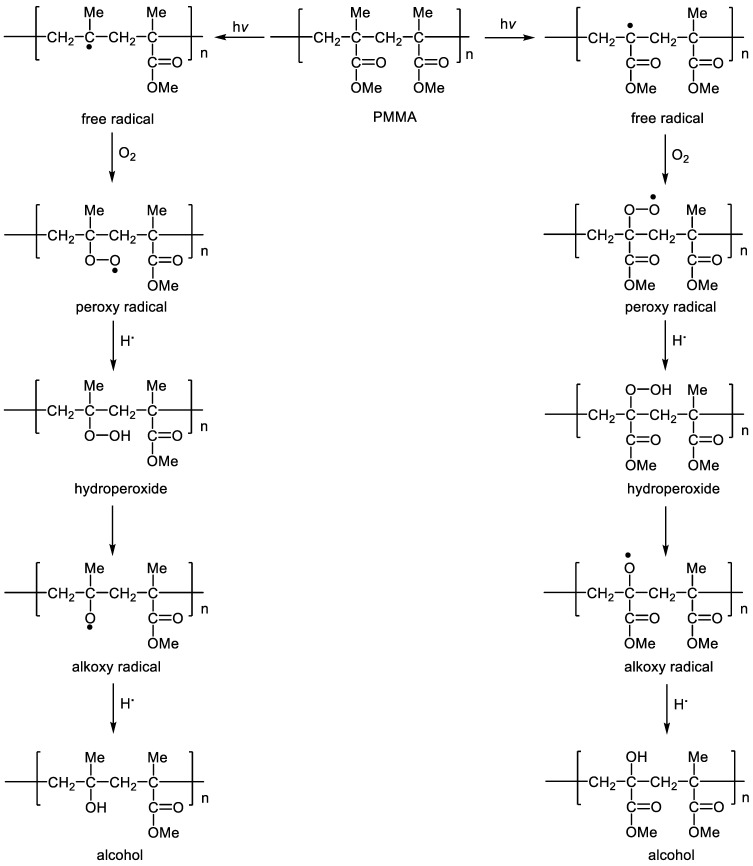
Photodegradation of PMMA and formation of fragments containing the OH group.

Photochemical degradation of PMMA leads to fragments that contain specific functional groups (e.g., hydroxy and carbonyl). The process involves the production of free radicals through the homolytic cleavage of the C–Me and C–CO_2_Me bonds (Figure 1). These free radicals can react with oxygen to yield the corresponding peroxy radicals. The peroxy radicals attached to the polymer can abstract hydrogen radicals from other polymeric chains to produce the corresponding hydroperoxides. The alkoxy radicals attached to the polymer are produced from the homolytic cleavage of the hydroperoxide O–OH bonds. The extraction of hydrogen radicals by alkoxy species leads to forming the corresponding alcohols (Figure 1).

**Figure 2:**
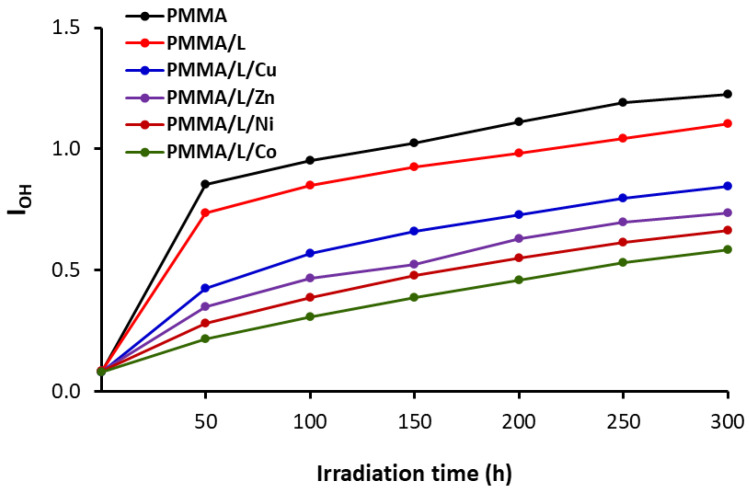
Hydroxyl index (I_OH_) of PMMA films during the irradiation process. L represents the ligand (Schiff base).

Thin films of PMMA were made and irradiated with UV light, and the FTIR spectra were recorded. The changes in the intensity of the peak corresponding to the stretching vibration of the OH group (3204 cm^–1^) were monitored [57] and compared to that of a reference peak (C–H or C–C). The increase in the intensity of the OH absorption band during irradiation reflected the degree of PMMA photodegradation. The I_OH_ was calculated for each modified PMMA using Equation (1) and plotted against irradiation time (Figure 2).

Increases in the I_OH_ were sharp at the beginning of irradiation, followed by a slower but gradual increase as time progressed. The highest rise in I_OH_ was seen for the unmodified PMMA film. In comparison, changes in I_OH_ were lower for the PMMA modified using Schiff base (L). The lowest changes in I_OH_ were observed for the PMMA films modified using Schiff base metal complexes. Thus, the aromatic moiety, heteroatoms, CH=N bonds, and metals must have played a role in reducing the photodegradation of the PMMA. At the end of the irradiation process, the I_OH_ was 1.22, 1.10, 0.85, 0.74., 0.66, and 0.58 for the unmodified PMMA, PMMA/Schiff base, PMMA/Schiff base/Cu, PMMA/Schiff base/Zn, PMMA/Schiff base/Ni, and PMMA/Schiff base/Co blends, respectively. The stabilization level depended on the metal’s identity, with their effectiveness following the order Co > Ni > Zn > Cu. It is not clear why Co led to the highest PMMA stabilization.

### 3.3. Weight Loss of Irradiated PMMA Films

**Figure 3:**
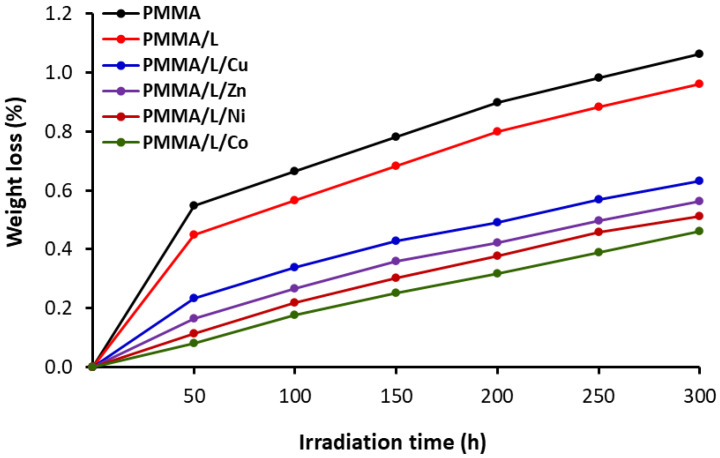
Weight loss (%) for PMMA films during the irradiation process. L represents the ligand (Schiff base).

Photodegradation of polymers leads to the production of small volatile fragments due to bond cleavage [58]. The weight loss (%) can be used as an indicator of the degree of PMMA photodegradation. Therefore, we investigated modified PMMA films’ photostability using weight loss. The PMMA films were irradiated, and the weight loss was calculated using Equation (2) from their weight pre- and post-irradiation. The weight loss (%) was plotted against the time of irradiation (Figure 3). The results were consistent with those obtained from FTIR spectroscopy reported in Section 3.2. As irradiation time increased, the weight loss increased, and was most apparent in the case of unmodified PMMA film. After 300 h of continuous irradiation, the weight loss (%) was 1.06, 0.96, 0.63, 0.56, 0.51, and 0.46 for the blank PMMA, PMMA/Schiff base, PMMA/Schiff base/Cu, PMMA/Schiff base/Zn, PMMA/Schiff base/Ni, and PMMA/Schiff base/Co blends, respectively.

### 3.4. Photodecomposition Rate Constant of PMMA

**Figure 4:**
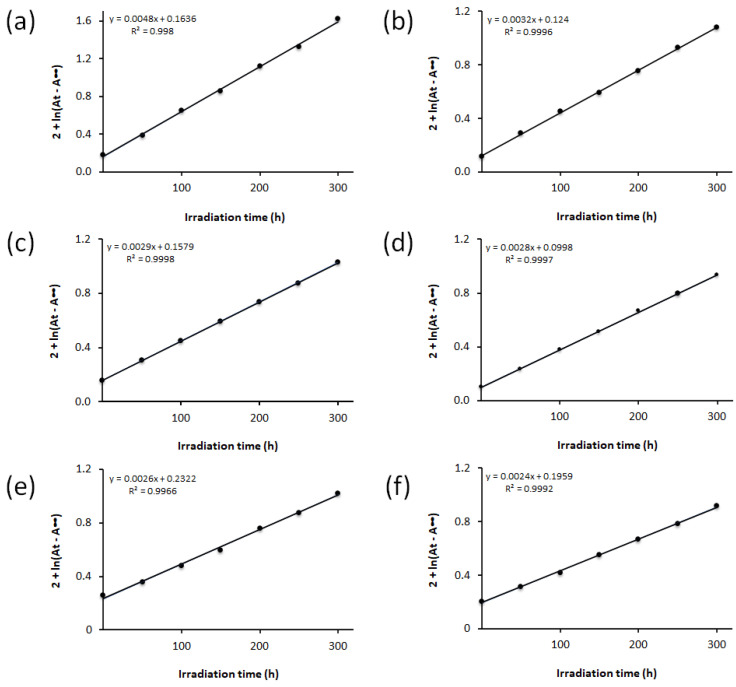
Changes in (*A_t_* − *A*_∞_) versus UV irradiation time for irradiated PMMA films: (**a**) PMMA, (**b**) PMMA/Schiff base, (**c**) PMMA/Schiff base/Cu, (**d**) PMMA/Schiff base/Zn, (**e**) PMMA/Schiff base/Ni, and (**f**) PMMA/Schiff base/Co.

The effect of pendant medications on the photodegradation of PMMA was investigated further by measuring their photodecomposition rate constants (K_d_) [59]. Table 2 summarises the K_d_ of PMMAs. Figure 4 shows the relationship between ${ln(A_{t}-A}_{\infty })$ and irradiation time (*t*). The plots gave straight lines with a slope equal to *k_d_*, indicating first-order kinetics for PMMA photodecomposition.

It was noted that all films decomposed during the photoirradiation process. The modified PMMA films had a lower K_d_ than the blank PMMA materials. The decomposition rate was highest for the unmodified film (4.8 × 10^–3^ s^–1^) and lowest for the polymer modified by incorporating Schiff base and cobalt as a metal (2.4 × 10^–3^ s^–1^). Clearly, the use of a combination of Schiff base and a metal (particularly cobalt) significantly enhanced PMMA photostabilization. Again, the results obtained from the measurements of K_d_ were consistent with those obtained from FTIR spectroscopy (Section 3.2) and weight loss determination (Section 3.3) for the PMMA films.

### 3.5. Surface Morphology Analysis

**Figure 5:**
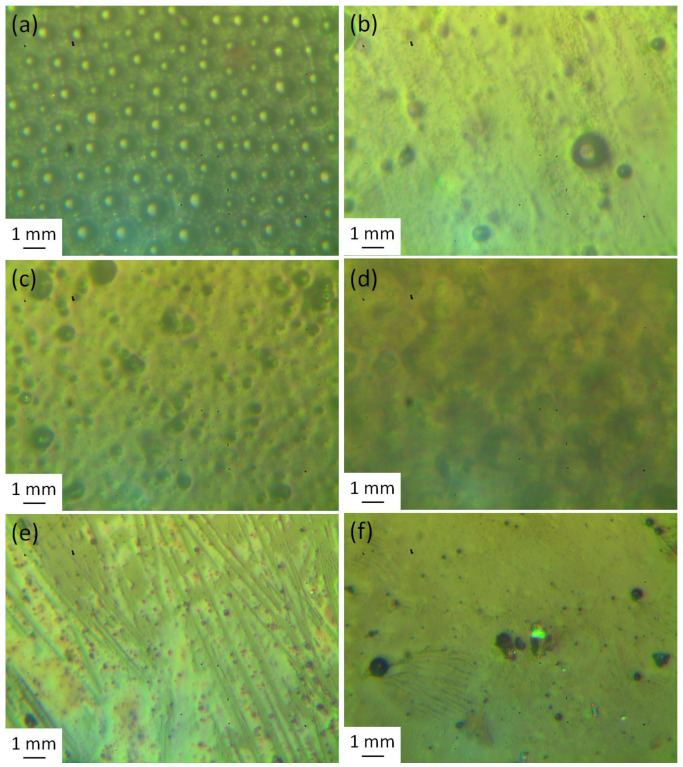
Optical images (400× magnification) of irradiated PMMA films: (**a**) PMMA, (**b**) PMMA/Schiff base, (**c**) PMMA/Schiff base/Cu, (**d**) PMMA/Schiff base/Zn, (**e**) PMMA/Schiff base/Ni, and (**f**) PMMA/Schiff base/Co.

Irradiation of PMMA films leads to defects and damage within the film’s surface. The surface morphology of the irradiated PMMA films was examined using various microscopic techniques [60,61,62,63,64,65]. The optical microscopy images of the PMMA reveal information about surface irregularity, roughness, and defects (e.g., dark spots, cracks, and grooves). Non-irradiated blank PMMA polymeric film (Figure S7) or those modified with Schiff bases and metals were smooth, undamaged surfaces with no holes, cracks, or black spots with no noticeable variations [66]. Figure 5 shows that irradiation caused damage (e.g., formation of dark spots and cracks along with discoloration) on the surfaces of the PMMAs. The surface of the unmodified PMMA (after irradiation (300 h)) showed many spots with a range of sizes. Generally, the PMMAs modified using metal complexes showed fewer spots than the unmodified film. This indicates that modification of the PMMA pendant led to a reduction in the photodegradation rate.

**Figure 6:**
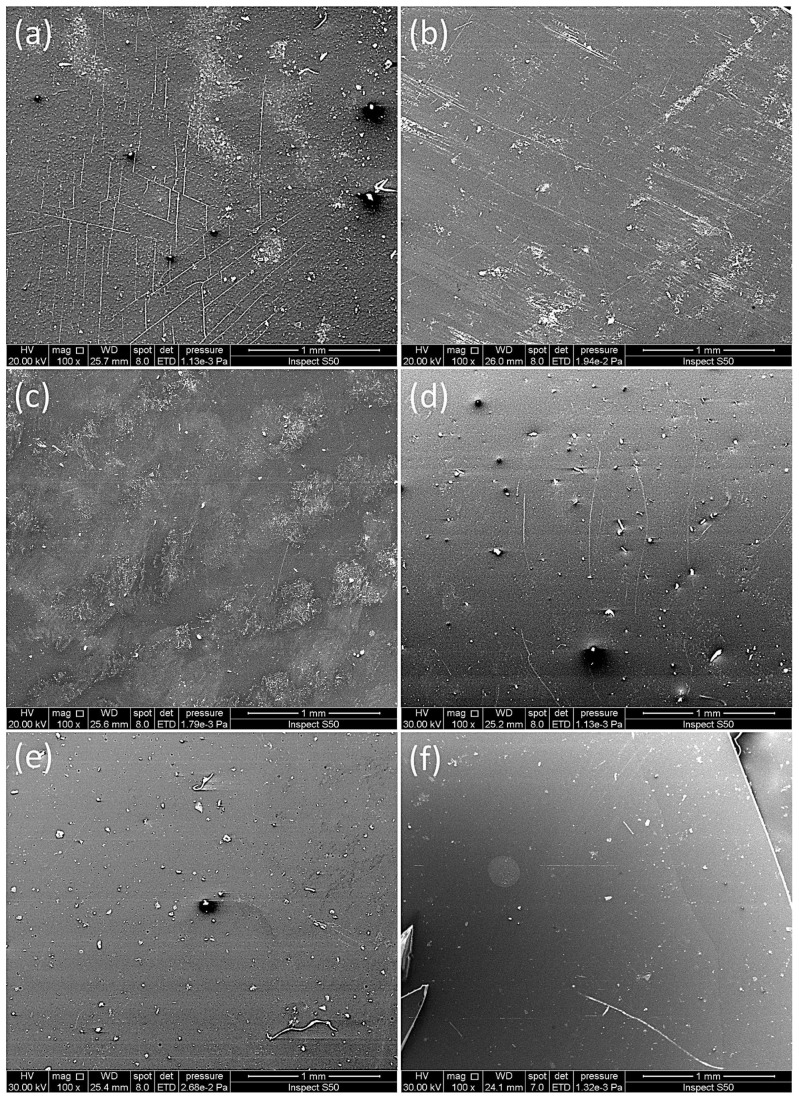
SEM images of irradiated PMMA films: (**a**) PMMA, (**b**) PMMA/Schiff base, (**c**) PMMA/Schiff base/Cu, (**d**) PMMA/Schiff base/Zn, (**e**) PMMA/Schiff base/Ni, and (**f**) PMMA/Schiff base/Co.

The SEM technique provides high-resolution undistorted images, and can be used as a reliable method to inspect the surfaces of materials. The SEM image for the non-irradiated PMMA film showed as smooth a surface (Figure S8) as the modified ones. Here, SEM images were used to assess the irregularity and homogeneity of the PMMAs’ surfaces. In addition, they facilitated a rough assessment of particle size, shape, pore diameters, and the appearance of grooves. SEM images of the PMMA films after irradiation are shown in Figure 6. Generally, weight loss from PMMA during irradiation leads to a damaged surface. However, the damage was significantly less noticeable for modified PMMAs. The least noticeable damage was apparent in the film containing cobalt.

**Figure 7:**
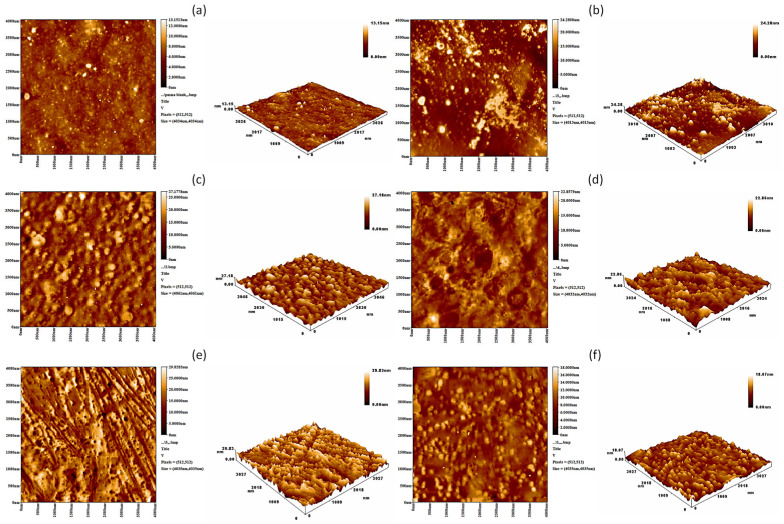
AFM images of irradiated PMMA films: (**a**) PMMA, (**b**) PMMA/Schiff base, (**c**) PMMA/Schiff base/Cu, (**d**) PMMA/Schiff base/Zn, (**e**) PMMA/Schiff base/Ni, and (**f**) PMMA/Schiff base/Co.

Finally, the surface of irradiated PMMA was inspected using two- and three-dimensional AFM images. AFM is a powerful method for assessing surface damage due to photoirradiation, as no vacuum is needed. Figure 7 shows that the surfaces of the irradiated PMMA films were rough. However, the roughness was lower for the modified PMMA films compared to the unmodified polymer. Table 3 shows that the roughness factor (Rq; nm) was highest for the unmodified PMMA film (285.1 nm) and lowest for the blend containing Schiff base and Co (64.9 nm).

### 3.6. Photostabilization Proposed Mechanisms

UV additives are important for protecting polymeric materials against UV irradiation [67]. Efficient UV photostabilizers act as UV absorbers, can generate electrons and holes at a high rate, and can scatter light. The UV light absorbed by photostabilizers can be remitted as energy at a less harmful wavelength to the materials, such as in the form of heat. The modification of PMMA by forming Schiff base moiety on the polymeric chains enabled UV light stabilization. The Schiff base contains heteroatoms (oxygen and nitrogen), the aromatic moiety (aryl ring), and the CH=N bond, which can absorb UV irradiation directly. In addition, modification enabled the PMMA films to contain metals that act as scavengers for hydroperoxides, peroxides, and radicals [68]. Therefore, the Schiff base metal complexes incorporated on the polymeric chains were capable of reducing the photodegradation of PMMA. The most noticeable stabilizing effect was seen for cobalt metal. It is unclear why the film containing cobalt was more photostabilized against irradiation than the others. The particle size of metals affects the absorption of UV irradiation [69]. However, UV light absorption is not the only factor that determines the efficiency of reducing PMMA photodegradation. A future study is needed to investigate the mechanism by which metals, particularly cobalt, are efficient in the photostabilization of polymers.

## 4. Conclusions

The surface modification of poly(methyl methacrylate), in which Schiff base metal complexes were incorporated in the polymeric chains, was successful. Modifying poly(methyl methacrylate) made it possible to minimize the harmful impact of ultraviolet irradiation (at a wavelength of 365 nm) for an extended period of up to 300 h. A significant improvement in the polymer’s photostabilization was noticed for the modified films compared to the unmodified film. The least destructive changes in the surface were seen in the poly(methyl methacrylate) containing Schiff base and cobalt, followed by those containing nickel, zinc, and copper. In addition, the surface modification of poly(methyl methacrylate) led to a noticeable reduction in the photodecomposition rate of polymeric materials. Both Schiff base and metals played roles in the photostabilization of the polymer. The Schiff base acted as an ultraviolet absorber, and the metals acted as free radical scavengers. However, the mechanism for the efficiency of the metals and Schiff base in stabilizing polymer against irradiation needs to be investigated.

## Figures and Tables

**Table 1:** Physical properties of PMMA materials.

Polymer	Color	Conversion (%)	M.P (°C)
PMMA	White	77	160–162
PMMA/Schiff base	Pale yellow	70	120–122
PMMA/Schiff base/Cu	Brown	72	280–282
PMMA/Schiff base/Zn	Yellow	74	264–266
PMMA/Schiff base/Ni	Yellow	71	280–282
PMMA/Schiff base/Co	Brown	70	220–222

**Table 2:** Rate of photodecomposition constant (k_d_) of PMMA films irradted with a UV light (λ_max_ = 365 nm) for 300 h.

Film	*k*_d_ (s^−1^)
PMMA	4.8 × 10^–3^
PMMA/Schiff base	3.2 × 10^–3^
PMMA/Schiff base/Cu	2.9 × 10^–3^
PMMA/Schiff base/Zn	2.8 × 10^–3^
PMMA/Schiff base/Ni	2.6 × 10^–3^
PMMA/Schiff base/Co	2.4 × 10^–3^

**Table 3:** Roughness factor (Rq; nm) of PMMA films after irradiation (300 h).

Film	Rq (nm)
PMMA	285.1
PMMA/Schiff base	115.8
PMMA/Schiff base/Cu	93.6
PMMA/Schiff base/Zn	90.4
PMMA/Schiff base/Ni	75.5
PMMA/Schiff base/Co	64.9

## Data Availability

Data presented in this study are available on request from the corresponding author.

## Supplementary Materials

The following supporting information can be downloaded at: https://www.mdpi.com/article/10.3390/polym15142989/s1, Figure S1: FTIR spectrum of unmodified PMMA, Figure S2: FTIR spectrum of PMMA/Schiff base, Figure S3: FTIR spectrum of PMMA/Schiff base/Cu, Figure S4: FTIR spectrum of PMMA/Schiff base/Zn, Figure S5: FTIR spectrum of PMMA/Schiff base/Ni, and Figure S6: FTIR spectrum of PMMA/Schiff base/Co, Figure S7: Optical image (400× magnification) of non-irradiated PMMA film, Figure S8: SEM image of non-irradiated PMMA film.
