# Supplementary material for: Pendant Modification of Poly(methyl methacrylate) to Enhance Its Stability against Photoirradiation
Source: Polymers (Basel). 2023 Jul 9;15(14):2989. doi: 10.3390/polym15142989 (PMC10386564; doi:10.3390/polym15142989)
Supplement: Supplementary file 1 [file polymers-15-02989-s001.zip › polymers-2470692-supplementary.pdf]

polymers

Supplementary Materials

Pendant Modification of Poly(methyl methacrylate) to Enhance its Stability against Photoirradiation

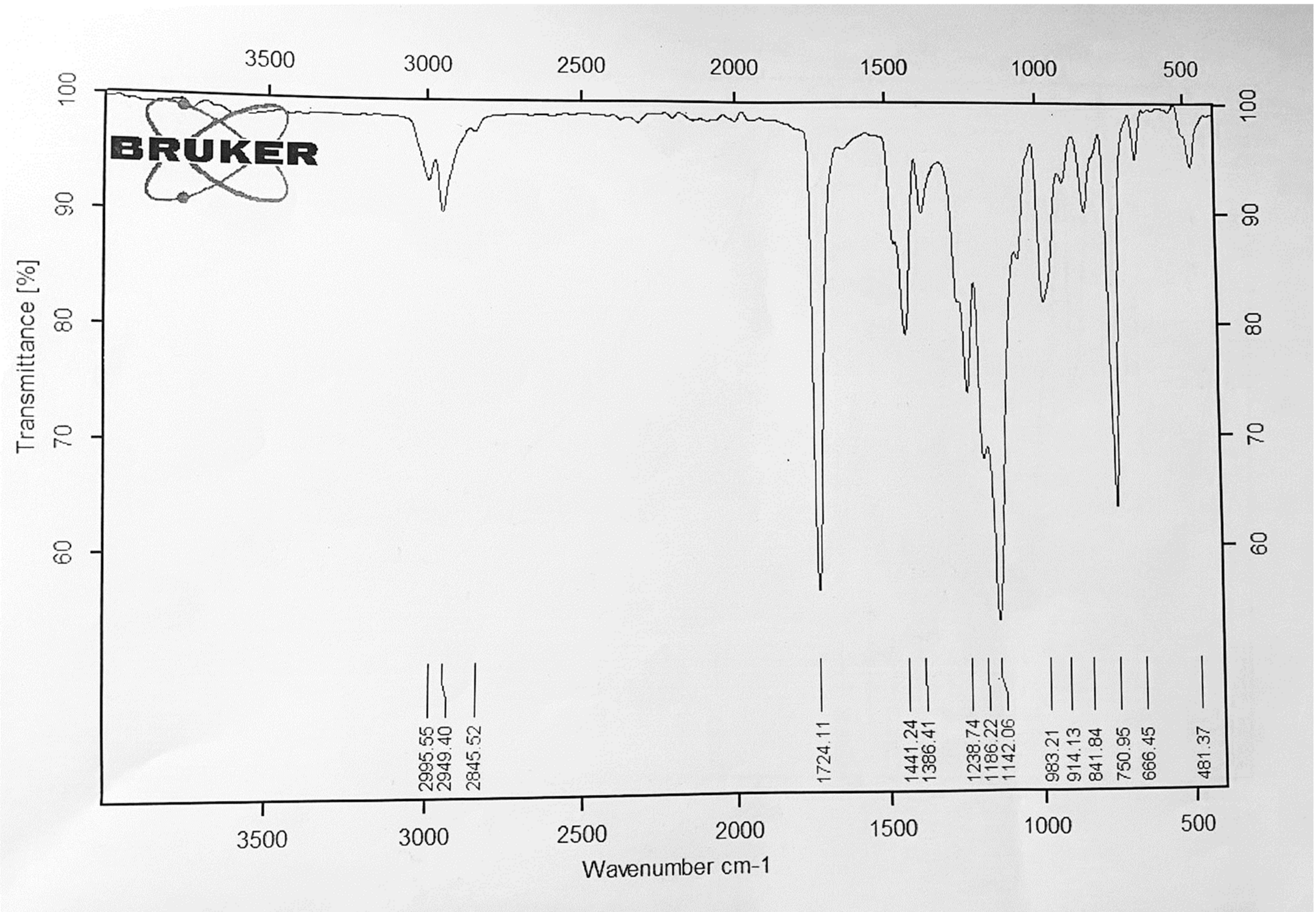

Figure S1. FTIR spectrum of unmodified PMMA.

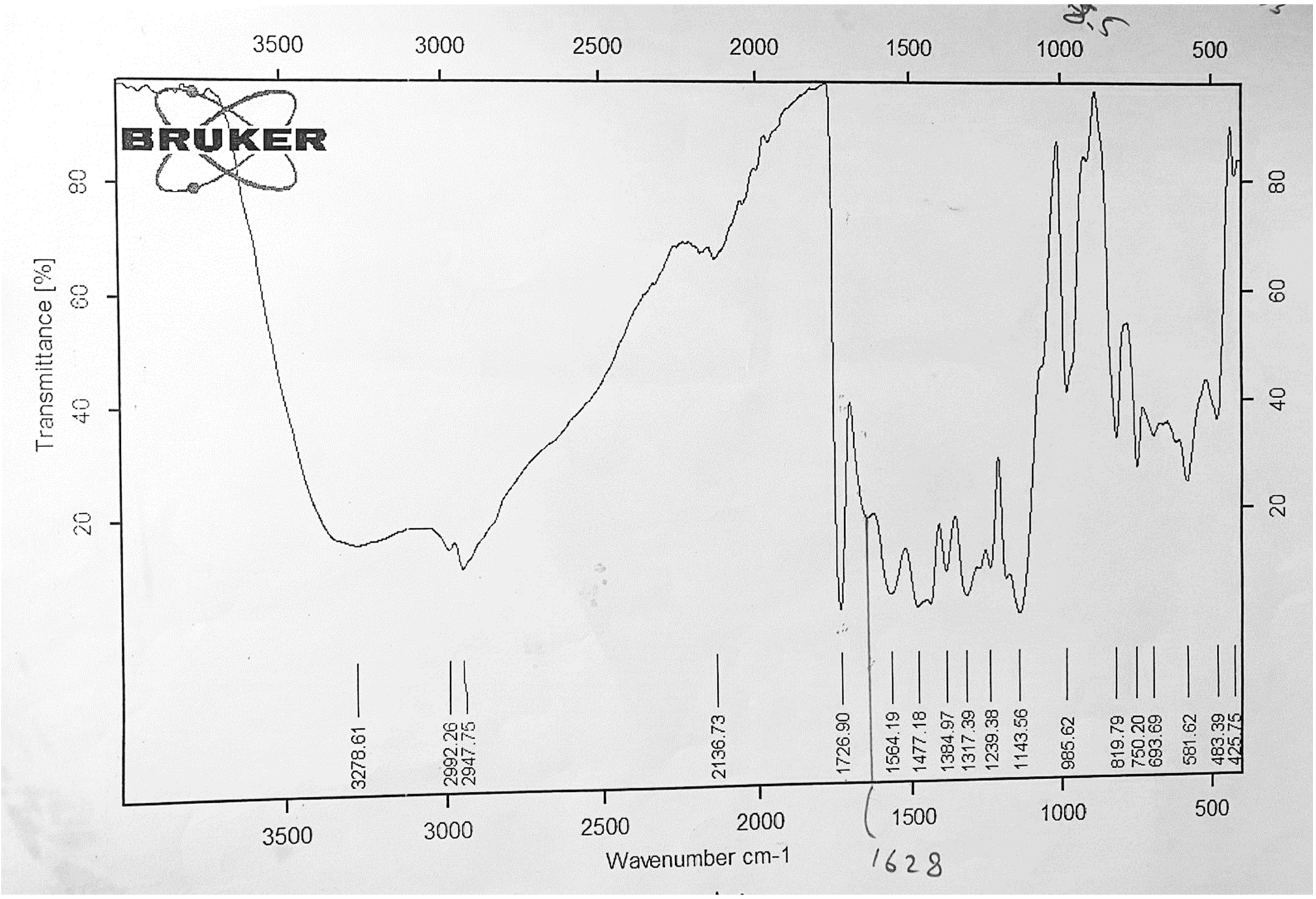

Figure S2. FTIR spectrum of PMMA/Schiff base.

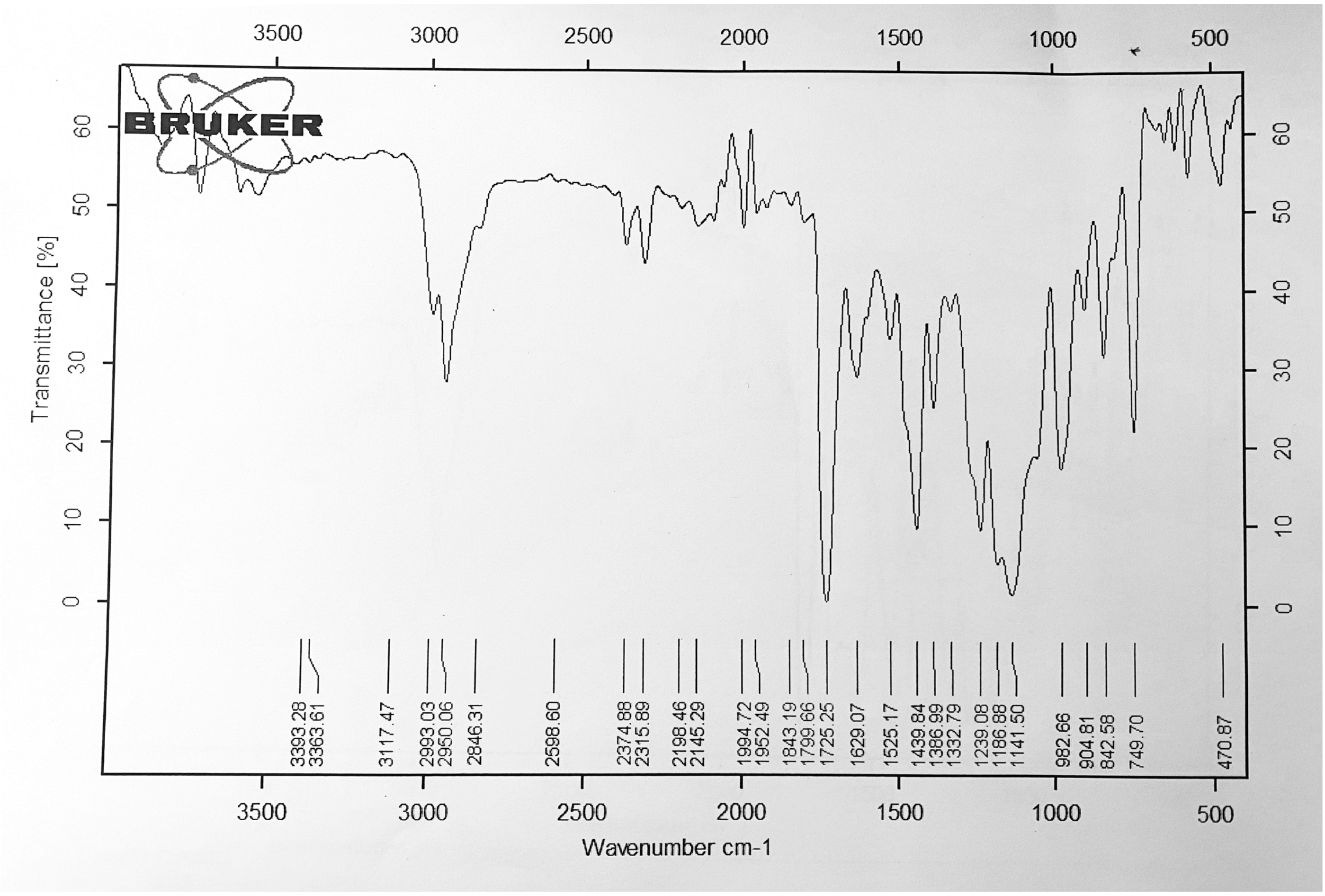

Figure S3. FTIR spectrum of PMMA/Schiff base/Cu.

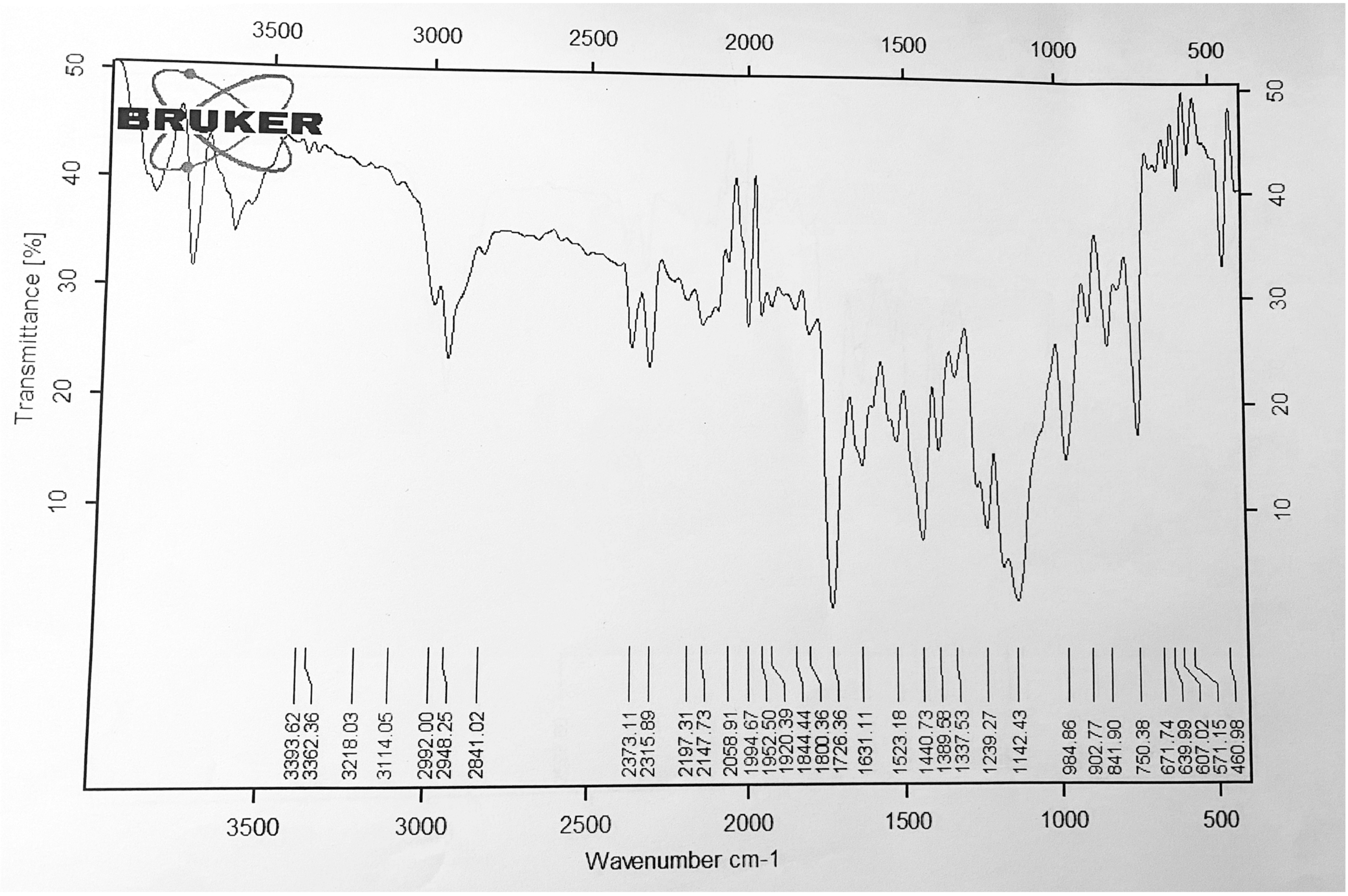

Figure S4. FTIR spectrum of PMMA/Schiff base/Zn.

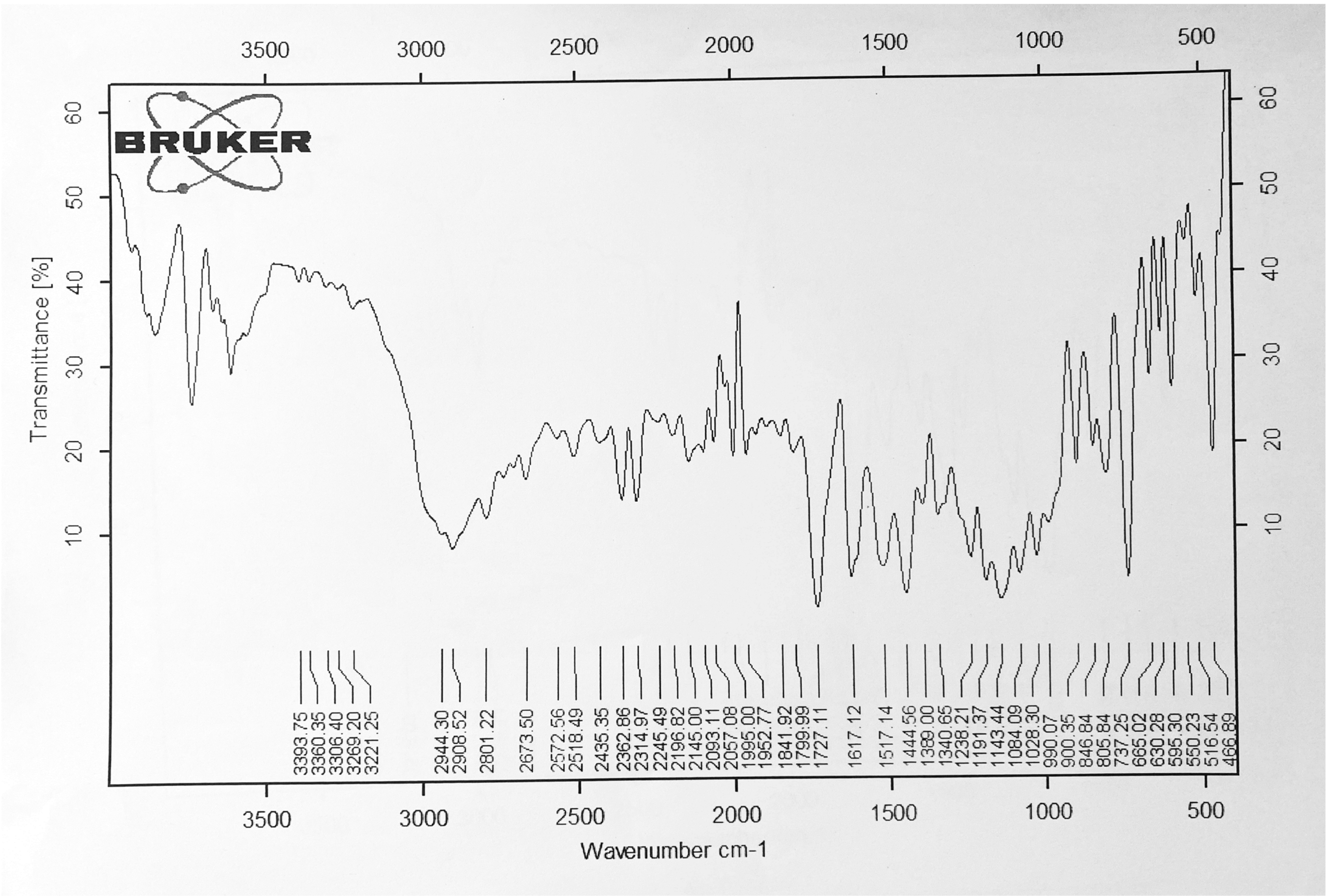

Figure S5. FTIR spectrum of PMMA/Schiff base/Ni.

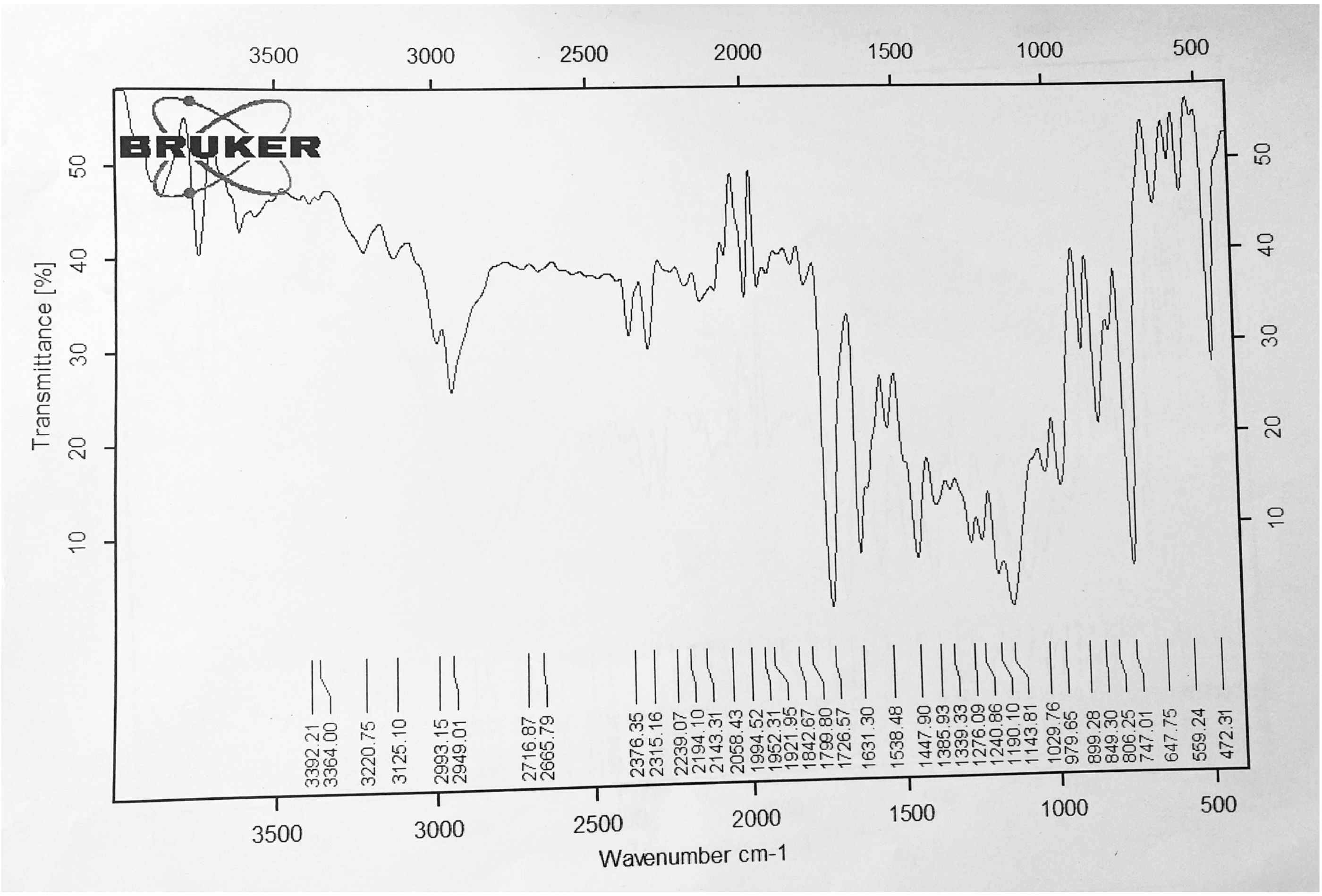

Figure S6. FTIR spectrum of PMMA/Schiff base/Co.

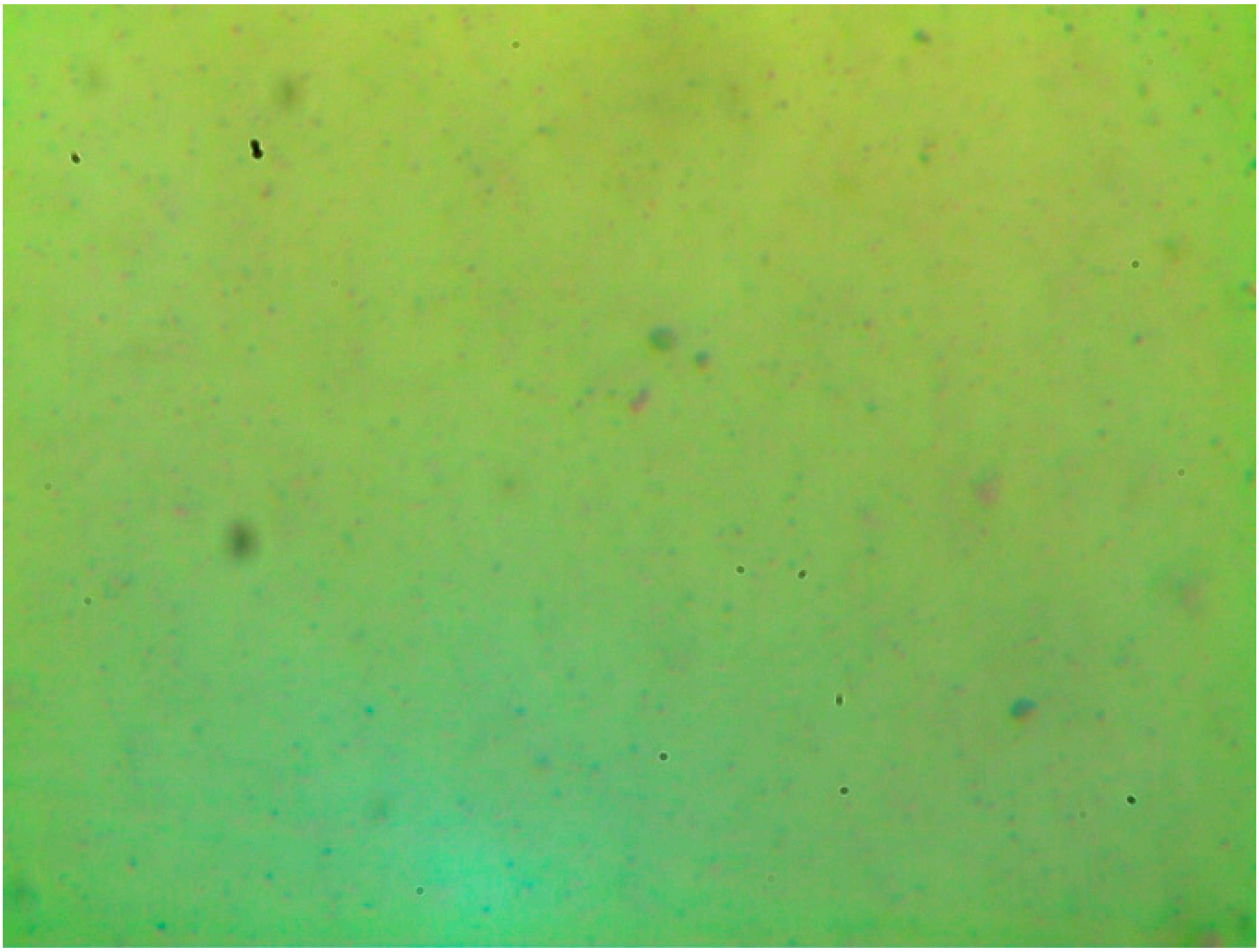

Figure S7. Optical image (400× magnification) of non-irradiated PMMA film.

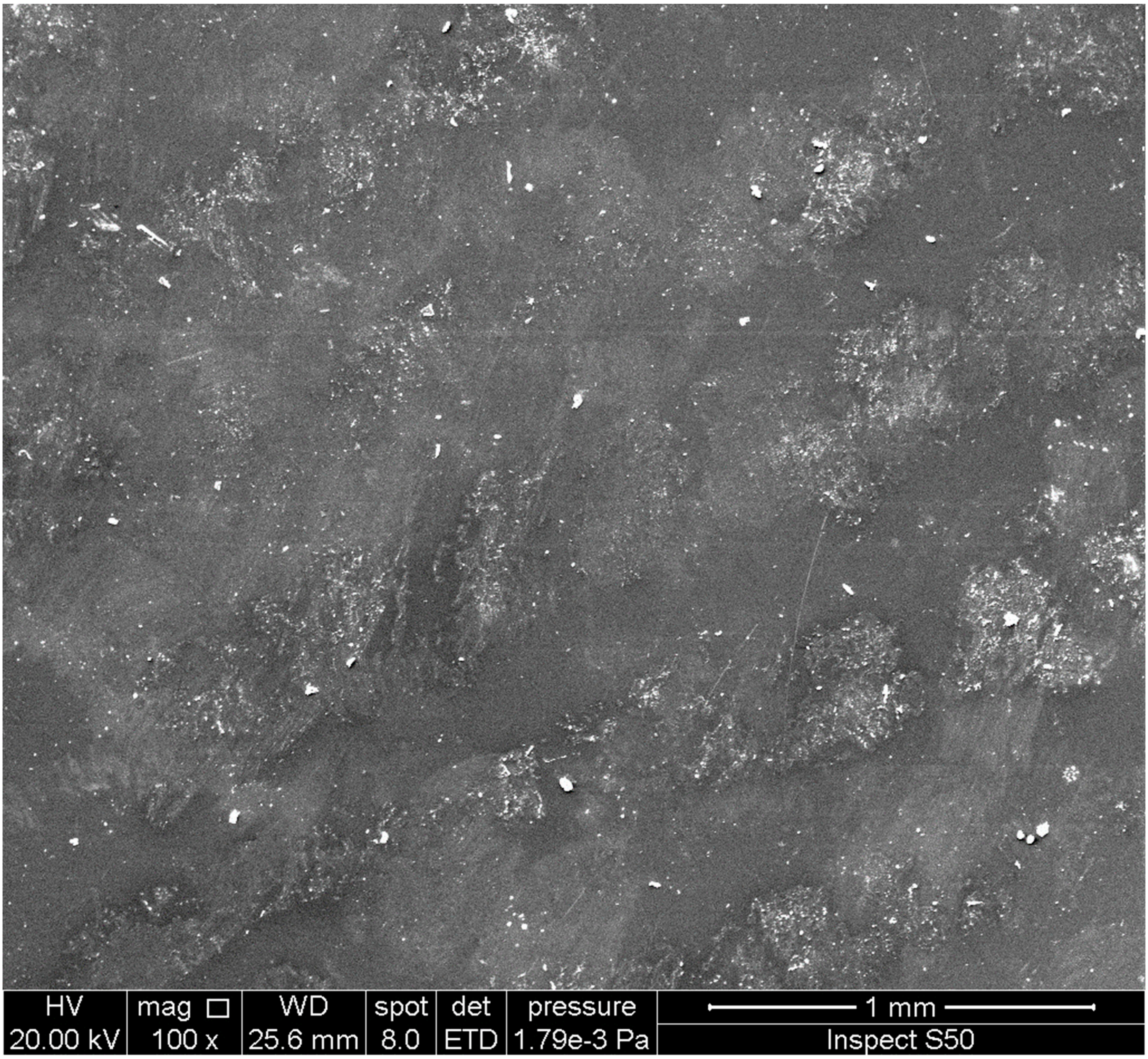

Figure S8. SEM image of non-irradiated PMMA film.
